# Statistics and data science unlock the predictive power of quantitative genetics

**DOI:** 10.3389/fpls.2026.1870471

**Published:** 2026-06-29

**Authors:** Osval A. Montesinos-López, José Crossa, Srikanth Kumar Karaikal, Leonardo Ornella, Joel A. Martínez-Regalado, Cinthia Leonora Murillo-Ávalos, Abelardo Montesinos-López, Iván Delgado-Enciso, Roberto de la Rosa Santa-María, Ezequiel Gamas-Alpuche, Verónica Miriam Guzmán-Sandoval, Andrés Eduardo Solís-Covarrubias, Nereyda C. Pérez-González, Karol D. Johnston Navarro, Mayte Muñoz-Rosales, Rodomiro Ortiz

**Affiliations:** 1 Facultad de Telemática, Universidad de Colima, Colima, Colima, Mexico; 2 Post-Graduate College (COLPOS) Montecillos, México, Mexico; 3 Plant Breeding Department. School of Integrative Plant Sciences, Section of Plant Breeding and Genetics, Cornell University, Ithaca, NY, United States; 4 Evogenix Ltd, London, United Kingdom; 5 Departamento de Estadística, Campus Miguel de Unamuno, Universidad de Salamanca, Salamanca, Spain; 6 Centro Universitario de Análisis Estadísticos y de Opinión Pública (CUOP), Universidad de Colima, Colima, Mexico; 7 Centro Universitario de Ciencias Exactas e Ingenierías (CUCEI), Universidad de Guadalajara, Jalisco, Mexico; 8 School of Medicine, University of Colima, Colima, Mexico; 9 Colegio de Postgraduados (CP), Campus Tabasco, Producción Agroalimentaria en el Trópico, H. Cárdenas, Tabasco, Mexico; 10 Facultad de Psicología, Universidad de Colima, Colima, Mexico; 11 Department of Plant Breeding, Swedish University of Agricultural Sciences (SLU), Lomma, Sweden

**Keywords:** big data, data science, data-driven methods, optimization, plant breeding, quantitative genetics, statistics

## Abstract

Plant breeding is undergoing a profound transition from an empirical and phenotype-centered discipline toward a predictive, inferential, and decision-oriented science. This transformation has been driven not only by the generation of large-scale genomic, phenotypic, environmental, pangenomic, and multi-omics datasets, but also by the statistical and data science methodologies required to convert such information into actionable breeding decisions. Quantitative genetics remains the theoretical foundation for understanding genetic variance, heritability, genotype-by-environment (*G*×*E*) interactions, breeding values, and response to selection; however, its practical implementation in modern breeding increasingly depends on high-dimensional modeling, uncertainty quantification, data integration, and computational prediction. In this review, we synthesize how statistics and data science expand the predictive potential of quantitative genetics through genomic selection, genome-wide association studies, mixed models, multivariate analysis, machine learning (ML), hypothesis testing, probability computation, visualization, and optimization. We emphasize that these tools should not be viewed as isolated analytical procedures, but as interconnected components of a breeding decision pipeline that supports parent selection, genotype advancement, trait prioritization, environmental targeting, training population design, resource allocation, and risk-aware selection under uncertainty. We also highlight key challenges, including the “large p, small n” problem, population structure, linkage disequilibrium, *G*×*E* interactions, non-additive genetic effects, overfitting, model interpretability, computational scalability, and the integration of heterogeneous data sources. Future progress will depend on developing integrated, interpretable, probabilistic, and scalable frameworks that combine genomic, phenotypic, environmental, pangenomic, and multi-omics information while remaining connected to biological meaning and practical breeding objectives. We conclude that statistics and data science do not replace quantitative genetics; rather, they operationalize and extend its principles, unlocking its predictive potential for more efficient, sustainable, and decision-oriented plant breeding.

## Introduction

Plant breeding is undergoing a profound transformation driven by the dual pressures of ensuring global food security and adapting agricultural systems to climate change, resource limitations, and a rapidly growing population projected to approach 9 billion by 2050. Historically, plant breeding relied on empirical selection guided by phenotypic observations and biological intuition (Bernardo, 2002). While these approaches delivered substantial genetic gains throughout the twentieth century, their efficiency is increasingly constrained by the complexity of modern breeding targets, which involve polygenic traits, genotype-by-environment (*G × E*) interactions, non-additive genetic effects, and dynamic production systems (Alemu et al., 2024).

In this context, plant breeding has evolved from an observational discipline into a predictive and decision-oriented science, where the central objective is no longer solely to evaluate observed performance but to accurately predict the genetic potential of untested genotypes and optimize selection decisions under uncertainty. This paradigm shift has been enabled by the rapid expansion of high-throughput technologies, including dense molecular markers, whole-genome sequencing, high-throughput phenotyping platforms, and the systematic collection of environmental covariates (Kumar et al., 2024). These technologies have generated unprecedented volumes of high-dimensional data, fundamentally redefining both the opportunities and challenges of plant breeding.

At the core of this transformation lies quantitative genetics, which provides the theoretical framework linking genetic variation to phenotypic expression and selection response. Classical quantitative genetics formalizes how complex traits arise from the combined effects of many loci, environmental influences, and their interactions, and establishes key principles such as heritability, genetic variance, and response to selection (Falconer and Mackay, 1996; Lynch and Walsh, 1998). These principles remain fundamental in modern breeding, but their implementation has shifted from analytical expressions based on simplified assumptions to data-driven estimation using high-dimensional genomic, phenotypic, and environmental information. In this sense, statistical models used in genomic selection (GS), genome-wide association studies (GWAS), and multi-environment analysis can be viewed as extensions of quantitative genetic theory to complex, high-dimensional settings, where prediction, inference, and uncertainty quantification are performed simultaneously.

A defining characteristic of modern breeding data is the “large p, small n” problem, where the number of predictors—often genomic markers, environmental covariates, or omics-derived variables—far exceeds the number of observations. Combined with the complex architecture of agronomic traits, which are typically controlled by many loci with small effects and influenced by *G × E* interactions, this creates a challenging inferential and predictive setting. Extracting reliable biological signals from such data requires statistical methodologies capable of handling multicollinearity, controlling overfitting, quantifying uncertainty, and ensuring robust generalization across populations and environments.

Before genomic prediction or association mapping can be applied effectively, breeding datasets must first be characterized in terms of structure, relatedness, dimensionality, and quality. Multivariate methods such as principal component analysis (PCA), clustering, and genomic relationship analysis are essential for identifying genetic structure, relationships among genotypes, outliers, and latent patterns in high-dimensional genomic and phenotypic data (Jolliffe and Cadima, 2016; Crossa et al., 2017). These analyses are not merely descriptive. They directly influence the construction of training populations, the interpretation of genomic relationships, the control of confounding in GWAS, and the evaluation of model transferability across populations and environments. Thus, exploratory and multivariate statistical methods provide a necessary foundation for both GS and GWAS.

One of the most significant manifestations of this transformation is GS, which conceptualizes plant breeding explicitly as a prediction problem. By leveraging genome-wide marker information, GS enables the estimation of genomic predictions for individuals with marker data but incomplete or unavailable phenotypic information, thereby supporting early selection, shortening breeding cycles, and increasing genetic gain per unit time (Alemu et al., 2024; Hayes and Goddard, 2001). From a statistical perspective, GS involves high-dimensional modeling, careful model validation, and the integration of diverse data sources, including genomic, phenotypic, environmental, and increasingly multi-omics data. The adoption of ML and deep learning methods has further expanded the capacity to capture complex and nonlinear relationships within these data (Yoosefzadeh Najafabadi et al., 2023).

Complementing prediction, statistical methodologies also underpin the discovery of genetic determinants of complex traits through GWAS. By exploiting linkage disequilibrium in diverse populations, GWAS identifies statistical associations between molecular markers and phenotypes, providing insights into trait architecture and supporting marker-assisted selection (Gangurde et al., 2022). However, because population structure and relatedness can generate spurious marker–trait associations, the prior characterization of genetic structure and the use of appropriate statistical corrections are essential for valid inference. The high-dimensional nature of genomic data further requires rigorous control of multiple testing and confounding effects, highlighting the central role of statistical reasoning in ensuring reliable biological interpretation.

Importantly, the genetic architecture underlying complex traits is often nonlinear, involving epistatic interactions among loci, dominance effects, and higher-order relationships with environmental variables. Classical linear models, while powerful and widely used, represent approximations to these underlying processes and may capture only part of the total genetic variation. This recognition has motivated the development of nonlinear statistical and ML approaches—including kernel methods, Bayesian models, and deep learning architectures—that are better suited to modeling complex interaction-driven biological systems (Yoosefzadeh Najafabadi et al., 2023). Thus, modern plant breeding increasingly requires methodological frameworks that move beyond simple linear assumptions while retaining biological interpretability and statistical rigor.

Within this framework, statistics and data science emerge as central engines of modern plant breeding, providing the theoretical and computational foundation for prediction, inference, and decision-making. Statistical models enable the estimation of genetic effects, the prediction of unobserved phenotypes, and the rigorous testing of hypotheses, while data science tools facilitate the integration, processing, visualization, and interpretation of large and heterogeneous datasets. Together, these disciplines transform raw biological data into actionable knowledge that directly informs breeding strategies.

Beyond prediction and association, modern plant breeding relies heavily on statistical tools for data exploration, pattern recognition, experimental design, and model evaluation. Descriptive statistics, visualization, and graphical diagnostics help assess data quality, detect outliers, evaluate residual patterns, and guide model choice. Additional tools such as heatmaps, dendrograms, Manhattan plots, quantile–quantile (QQ) plots, spatial field maps, and observed-versus-predicted plots enable the interpretation of complex genetic and phenotypic patterns (Jolliffe and Cadima, 2016; Crossa et al., 2017). These methods help prevent biased inference and support decisions regarding training population design, genotype classification, environmental targeting, and selection strategy.

As breeding programs increasingly integrate genomic, phenotypic, environmental, pedigree, and multi-omics data, the role of statistics and data science extends beyond analysis to encompass the entire decision-making pipeline. This includes optimizing the allocation of limited resources, evaluating uncertainty in predictions, computing probabilities of selecting superior genotypes, and determining how selection decisions should be made under varying environmental and genetic contexts (Piepho et al., 2008). In this sense, modern plant breeding can be viewed as a unified framework in which prediction, inference, uncertainty quantification, and optimization are tightly interconnected through statistical principles.

Importantly, the value of statistical and data science tools in plant breeding should ultimately be evaluated not only by their methodological sophistication or predictive accuracy, but by their ability to improve concrete decisions made by breeders. These decisions include which parents to cross, which genotypes to advance, which environments to target, which traits to prioritize, how training populations should be designed, and how limited resources should be allocated across phenotyping, genotyping, and multi-environment testing. Thus, prediction, inference, visualization, hypothesis testing, probability computation, and optimization are not isolated analytical exercises; they are interconnected components of a decision-making pipeline that transforms biological data into actionable breeding strategies.

Given these developments, this review synthesizes the central role of statistical and data science methodologies in contemporary plant breeding. Specifically, we examine their contributions to: (1) the prediction of unobserved genotypes using statistical and ML models; (2) the identification of significant marker–trait associations through GWAS; (3) the description and visualization of high-dimensional breeding datasets; (4) pattern recognition and dimensionality reduction for uncovering latent biological structure; (5) the optimization of limited resources in breeding programs; (6) hypothesis testing to support robust scientific inference and decision-making; and (7) the computation of probabilities associated with selection and genetic gain. By integrating these perspectives, we aim to provide a coherent framework that highlights how statistical and data science approaches drive innovation, efficiency, and better decision-making in modern plant breeding.

## Statistical and data science approaches in predictive plant breeding

### Predictions in plant breeding: foundations, advances, and challenges

**Figure 1:**
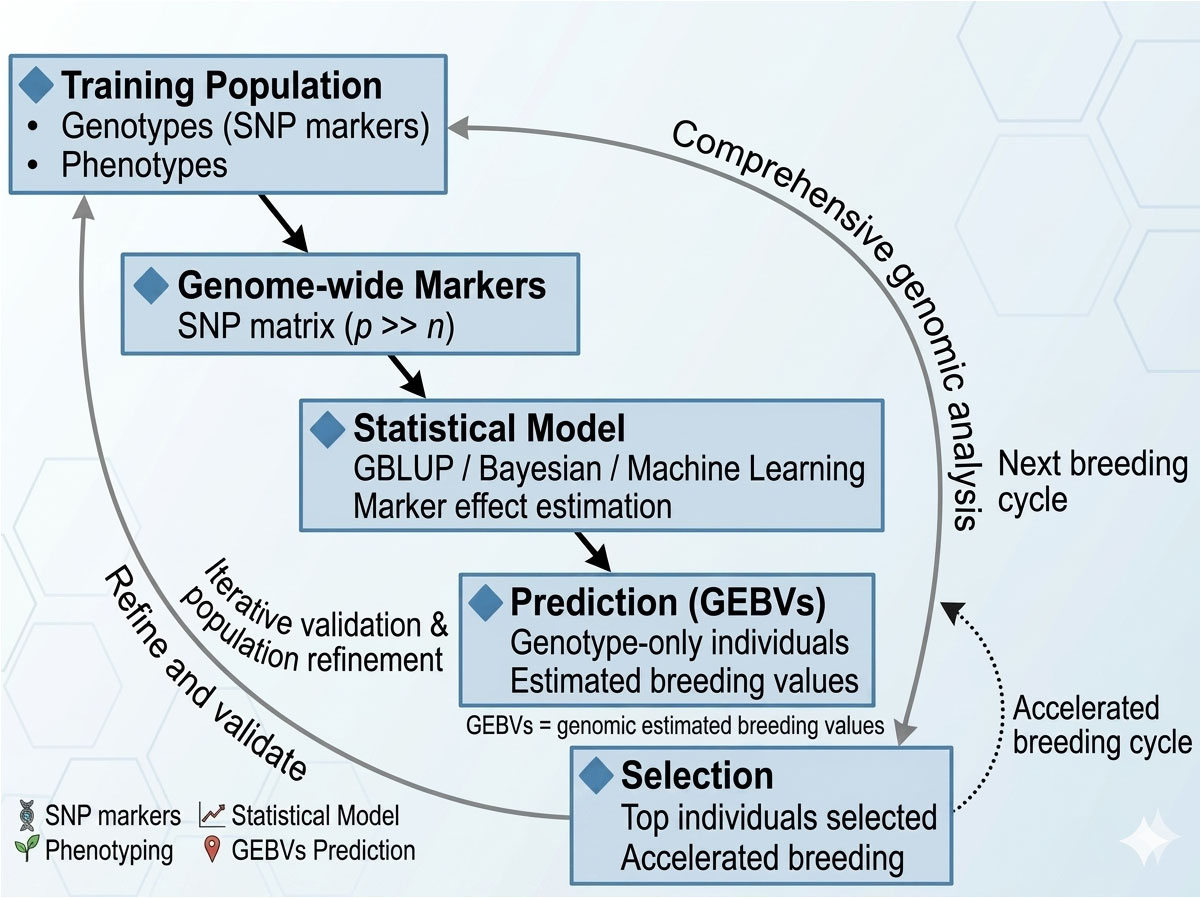
Conceptual workflow of genomic selection (GS) in plant breeding. Models trained on genotypic and phenotypic data from a training population are used to predict genomic estimated breeding values (GEBVs) for genotype-only individuals, enabling early selection and accelerating breeding cycles in an iterative process. Graphic design assistance provided by Gemini 3 Flash (Google).

One of the most transformative advancements in modern plant breeding is genomic selection (GS), a class of statistical approaches that uses genome-wide marker information to predict genetic merit and guide selection decisions before complete phenotypic evaluation is available. By training models on a reference or training population with both genotypic and phenotypic information, breeders can predict the performance of candidate genotypes that have marker data but incomplete or unavailable phenotypic records (**Figure 1**). This strategy can accelerate selection cycles, reduce phenotyping costs, and increase genetic gain per unit time, particularly when prediction accuracy is sufficiently high, and selection can be conducted before extensive multi-environment testing (Alemu et al., 2024; Hayes and Goddard, 2001; Meuwissen et al., 2001).

A clarification is important regarding the term genomic estimated breeding value (GEBV). In the classical quantitative genetic framework, breeding value refers primarily to the additive genetic component that is transmitted from parents to offspring. Therefore, GEBV usually denotes the additive genomic breeding value predicted from genome-wide markers. However, broader genomic prediction models may also be formulated to estimate total genetic values or specific non-additive components, including dominance, epistasis, and *G* × E interactions. This distinction is important because modern statistical and ML approaches are increasingly used not only to predict additive merit, but also to model more complex genetic architectures. Thus, throughout this review, we distinguish between additive GEBVs and broader genomic predictions of total or component-specific genetic effects.

At its core, GS represents a high-dimensional prediction problem in which the number of molecular markers typically exceeds the number of phenotyped individuals. This “large p, small n” setting requires statistical methodologies capable of handling multicollinearity, controlling overfitting, estimating genetic relationships, and ensuring robust prediction across populations and environments. A wide range of models has been developed for this purpose, including genomic best linear unbiased prediction (GBLUP), Bayesian regression models such as BayesA, BayesB, BayesCπ, Bayesian ridge regression, and Bayesian least absolute shrinkage and selection operator (LASSO), as well as penalized regression methods such as ridge regression, LASSO, and elastic net (Endelman and Jannink, 2012; Heslot et al., 2012; Jannink et al., 2010).

These methods differ in their assumptions about marker effects and genetic architecture. GBLUP assumes many small additive effects and is robust for highly polygenic traits, but it may not explicitly identify major loci or capture complex non-additive interactions. Bayesian variable-selection models can be useful when some markers have moderate or large effects, but they may be more computationally demanding and sensitive to prior assumptions. Penalized regression approaches provide flexible shrinkage and variable selection, but their performance depends on tuning and validation. Kernel-based methods, including reproducing kernel Hilbert space (RKHS) regression, can capture nonlinear genomic relationships and some forms of epistasis, whereas *ML* approaches such as random forests (*RF*), support vector machines (*SVMs*), and deep learning architectures may detect complex patterns but require careful validation to avoid overfitting and loss of biological interpretability (Heslot et al., 2012; Crossa et al., 2017; Manthena et al., 2022; Yoosefzadeh Najafabadi et al., 2023).

The practical success of GS depends not only on model choice but also on training population design, trait heritability, population structure, marker density, relatedness between training and candidate populations, and the target prediction scenario. In applied breeding programs, GS has been used to prioritize candidates for advancement, reduce the number of genotypes entering expensive multi-environment trials, and support earlier selection decisions. For example, in cereal breeding, genomic prediction has been used to screen large numbers of lines before complete field testing, while in recurrent selection schemes it can shorten the interval between cycles by enabling selection based on marker information. These applications illustrate the central practical value of GS: it improves breeder decisions by increasing the accuracy and timeliness of selection under limited resources (Alemu et al., 2024; Crossa et al., 2017).

Despite its considerable success, GS continues to face important challenges. Prediction accuracy may decline when the training and prediction populations are weakly related, when environmental conditions differ substantially, or when trait architecture involves strong *G × E* or non-additive effects not adequately represented by the model. The integration of multi-omics, high-throughput phenotyping, and environmental covariates can improve prediction in some contexts, but these additional data layers also introduce noise, missing values, dimensionality, and computational burden. Therefore, the adoption of complex models should be justified by clear improvements in prediction accuracy, biological interpretation, or breeding decisions, rather than by methodological complexity alone.

### Genome-wide association studies

Complementing prediction, statistics, and data science enable the discovery of genomic regions associated with complex traits through a GWAS. The GWAS, also known as association mapping, exploits historical recombination in diverse germplasm panels to identify statistical associations between molecular markers and phenotypes. In contrast to traditional linkage mapping based on biparental populations, GWAS can achieve higher mapping resolution because it uses recombination accumulated over many generations. However, this resolution depends strongly on marker density and the extent of linkage disequilibrium (LD) in the population under study (Yu et al., 2006; Korte and Farlow, 2013; Gangurde et al., 2022).

The role of LD is central to interpreting GWAS results. When LD decays rapidly, as often occurs in diverse or outcrossing populations, markers must be very dense to tag causal variants or genomic regions associated with the trait. In such cases, GWAS may achieve high resolution but requires extensive genotyping coverage. In contrast, when LD extends over long genomic regions, fewer markers may be sufficient to detect associations, but the associated regions may be large and contain many candidate genes, reducing mapping precision. Therefore, the apparent advantage of GWAS over linkage analysis depends not only on the use of diverse germplasm, but also on species biology, population history, recombination rate, marker density, and genomic architecture.

**Figure 2:**
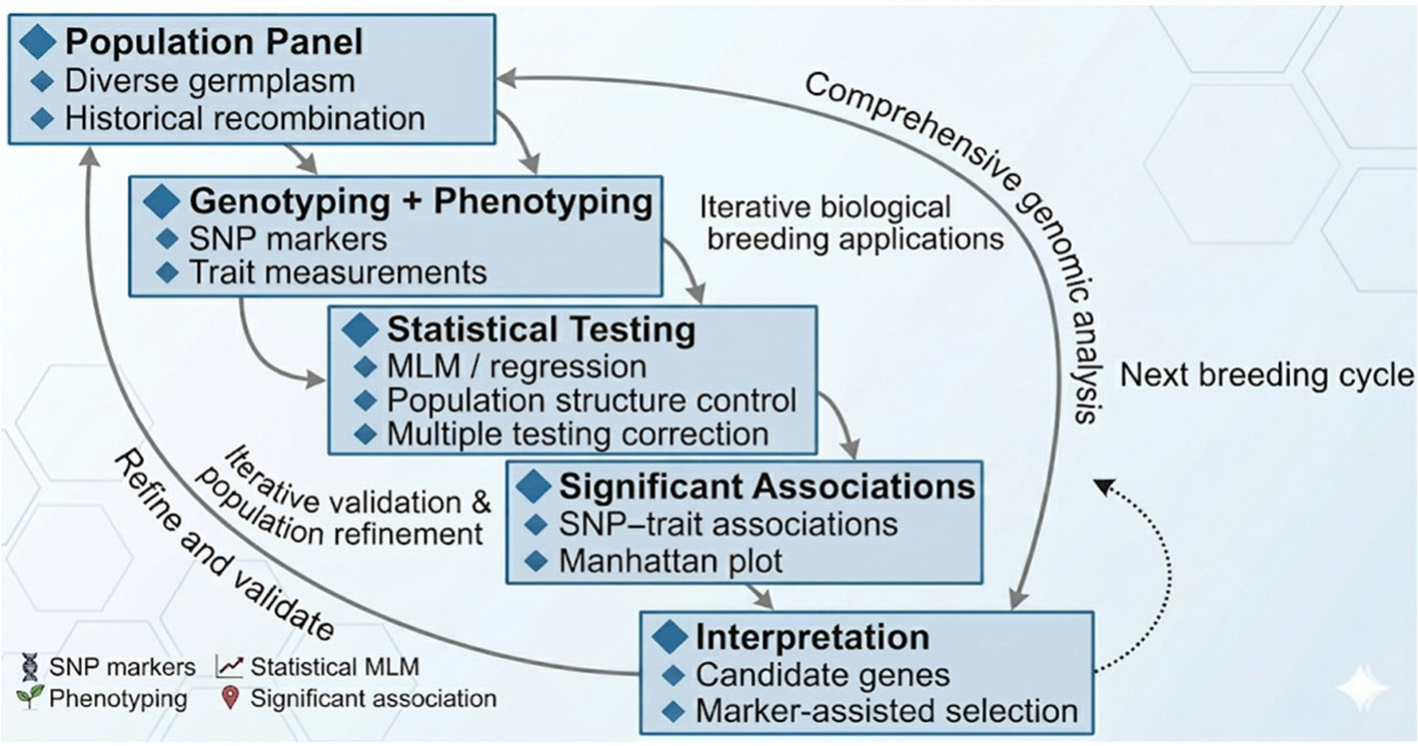
Conceptual workflow of genome-wide association studies (GWAS) in plant breeding. A diverse population panel is genotyped and phenotyped to enable statistical testing of marker–trait associations using regression or mixed linear models that account for population structure and multiple testing. Significant associations are identified and visualized (e.g., Manhattan plots), enabling the discovery of candidate genes and supporting marker-assisted selection. The process is iterative, incorporating validation and refinement to improve biological interpretation and breeding applications. Graphic design assistance provided by Gemini 3 Flash (Google).

At its core, GWAS relies on hypothesis testing within regression or mixed-model frameworks, where each marker or set of markers is tested for association with a phenotype (**Figure 2**). Simple single-marker general linear models (GLM) are easy to implement and interpret, but they are vulnerable to false positives when population structure and relatedness are not adequately controlled. Mixed linear models (MLM), which include kinship and population structure, represented a major advance because they reduce confounding and improve inference reliability (Yu et al., 2006). However, MLM approaches may also be conservative and can lose power when the tested markers are confounded with the kinship structure.

Multi-locus GWAS methods were developed to address some limitations of single-marker testing and overly conservative mixed models. These approaches can consider multiple associated markers simultaneously, reduce residual confounding, and improve the detection of loci with moderate effects. One widely used example is Fixed and random model Circulating Probability Unification (FarmCPU), which iteratively uses fixed-effect and random-effect models to control false positives while improving statistical power. FarmCPU separates the testing of markers from the optimization of pseudo-quantitative trait nucleotides, reducing confounding between population structure, kinship, and true marker effects. This makes it particularly useful in high-dimensional GWAS settings where both false positives and false negatives are major concerns (Liu et al., 2016).

Different GWAS methods therefore have different strengths and limitations. GLM approaches are simple but vulnerable to confounding; MLM approaches control structure and kinship but may reduce power; multi-locus approaches such as FarmCPU can improve detection but require careful implementation and validation; Bayesian and penalized regression approaches can be useful for complex architectures but may be computationally intensive and sensitive to assumptions. Consequently, GWAS results should be interpreted in light of population structure, LD, marker density, sample size, phenotypic quality, and environmental heterogeneity.

From a breeding perspective, GWAS contributes to decision-making by identifying candidate genomic regions, improving understanding of trait architecture, prioritizing markers for validation, and supporting marker-assisted selection for traits controlled by major loci. However, many agronomic traits are highly polygenic and influenced by G × E interactions, which limits the direct application of individual marker associations. Therefore, GWAS should not be viewed only as a marker-discovery tool, but also as a source of biological information that can complement genomic prediction.

Importantly, GWAS and GS are not isolated approaches. Genomic regions identified by GWAS can be incorporated into GS models as fixed effects, weighted markers, biological priors, or kernel components. Such integration may improve prediction accuracy and biological interpretability, particularly when traits are influenced by moderate- or large-effect loci. However, for highly polygenic traits, the advantage of adding GWAS-derived markers may be limited, and whole-genome prediction models that capture many small effects across the genome often remain more robust. The most useful strategy depends on trait architecture, population size, validation design, and the breeding decision being supported.

### Dataset description and visualization in plant breeding

Modern plant breeding generates vast and heterogeneous datasets that extend beyond simple phenotypic measurements. These datasets include genomic profiles from high-throughput platforms, multi-environment phenotypes, pedigree information, environmental covariates, field spatial information, and increasingly multi-omics layers such as transcriptomics and metabolomics. Effectively handling this complexity requires rigorous statistical tools capable of summarizing central tendencies, dispersion, distributional properties, missing values, outliers, and correlations among variables (Eraslan et al., 2019).

Descriptive statistics, including means, variances, medians, skewness, quantiles, and correlations, form the foundation for assessing data quality and ensuring reliable downstream inference. In breeding programs, these initial analyses are not merely preliminary; they influence decisions about whether trials are usable, whether traits require transformation, whether outliers should be investigated, and whether experimental precision is adequate for selection. Because breeding decisions often depend on detecting subtle genetic differences amid environmental noise, careful data description is essential for robust selection.

**Figure 3:**
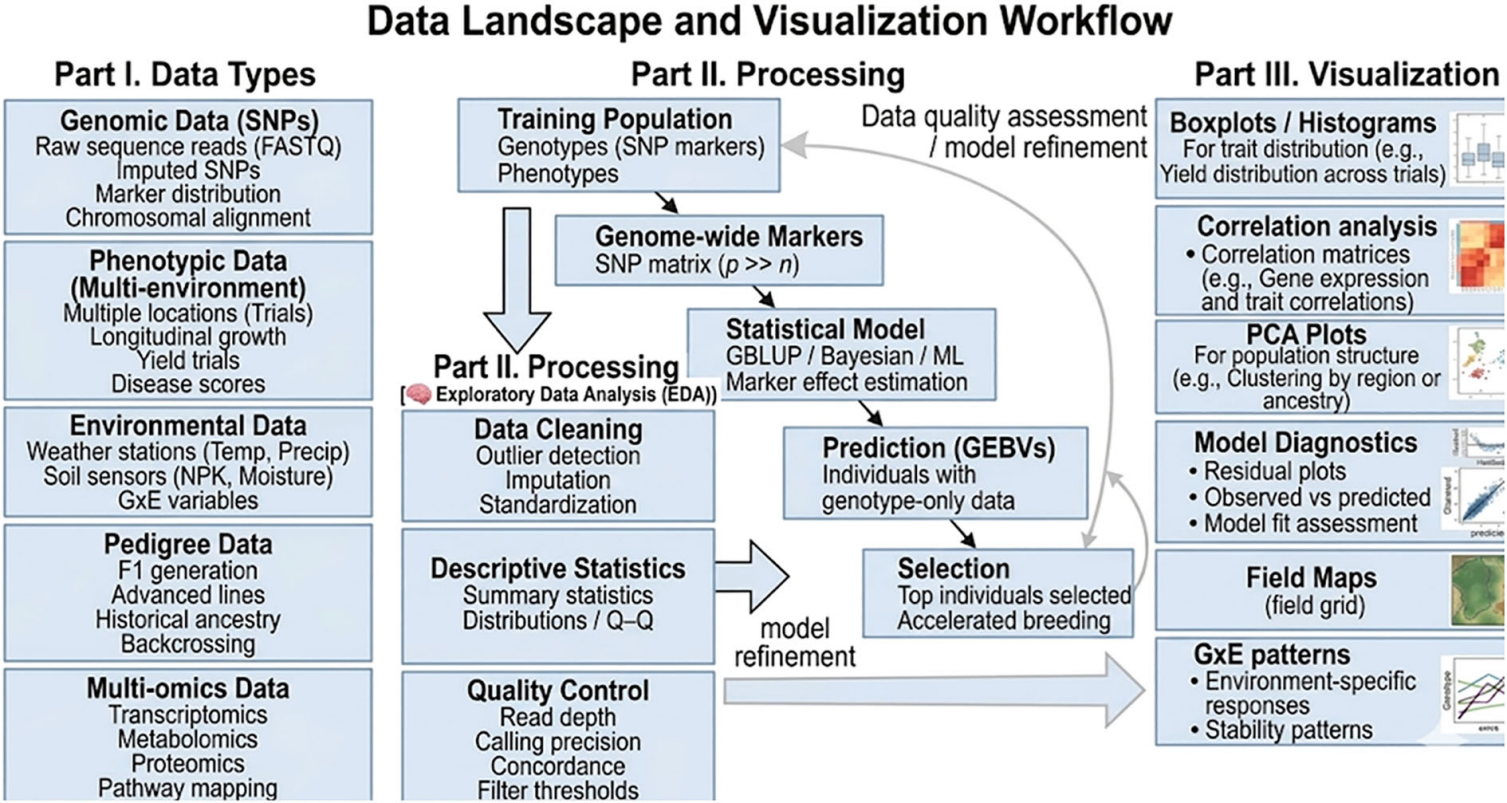
Conceptual framework for data characterization, preprocessing, and visualization in plant breeding, highlighting the integration of multi-source data (genomic, phenotypic, environmental, pedigree, and multi-omics), statistical modeling, prediction, and visualization to support selection decisions and iterative breeding cycles. Graphic design assistance provided by Gemini 3 Flash (Google).

Visualization techniques translate high-dimensional data into interpretable graphical representations that facilitate pattern recognition, diagnostic assessment, and communication with breeders. Tools such as boxplots, histograms, scatterplots, and correlation heatmaps reveal trait distributions, genotype relationships, and potential data issues, including heterogeneity of variance or measurement error (**Figure 3**). In genomic contexts, allele frequency plots, LD decay curves, genomic relationship matrices, and PCA plots help identify population structure, relatedness, and possible subgroups. Additional tools such as Manhattan plots, QQ plots, heatmaps, dendrograms, spatial field maps, time-series visualizations, observed-versus-predicted plots, and residual diagnostics support interpretation of complex genetic and phenotypic patterns (**Figure 3**).

The widespread adoption of R and Python has strengthened reproducible and scalable data analysis in plant breeding, with packages such as ggplot2 enabling flexible visualization and libraries such as pandas supporting efficient data manipulation (McKinney, 2010; Wickham et al., 2015; Wickham, 2016; R Core Team, 2026). However, visualization should not be treated as decoration. Its value lies in improving data quality control, model diagnosis, training population construction, environmental characterization, and selection decisions. Thus, descriptive analysis and visualization constitute essential components of the predictive breeding pipeline.

### Pattern recognition in plant breeding

Pattern discovery is a central analytical objective in modern plant breeding because it enables the identification of latent structures and relationships within genomic, phenotypic, environmental, and multi-omics datasets. Unlike purely confirmatory analyses, pattern recognition applies statistical and data-driven methods to uncover meaningful organization without relying exclusively on predefined hypotheses. These approaches help reveal genetic diversity, population structure, genotype-by-environment patterns, mega-environments, trait correlations, and multivariate signatures associated with adaptation or superior performance (Hastie et al., 2009).

**Figure 4:**
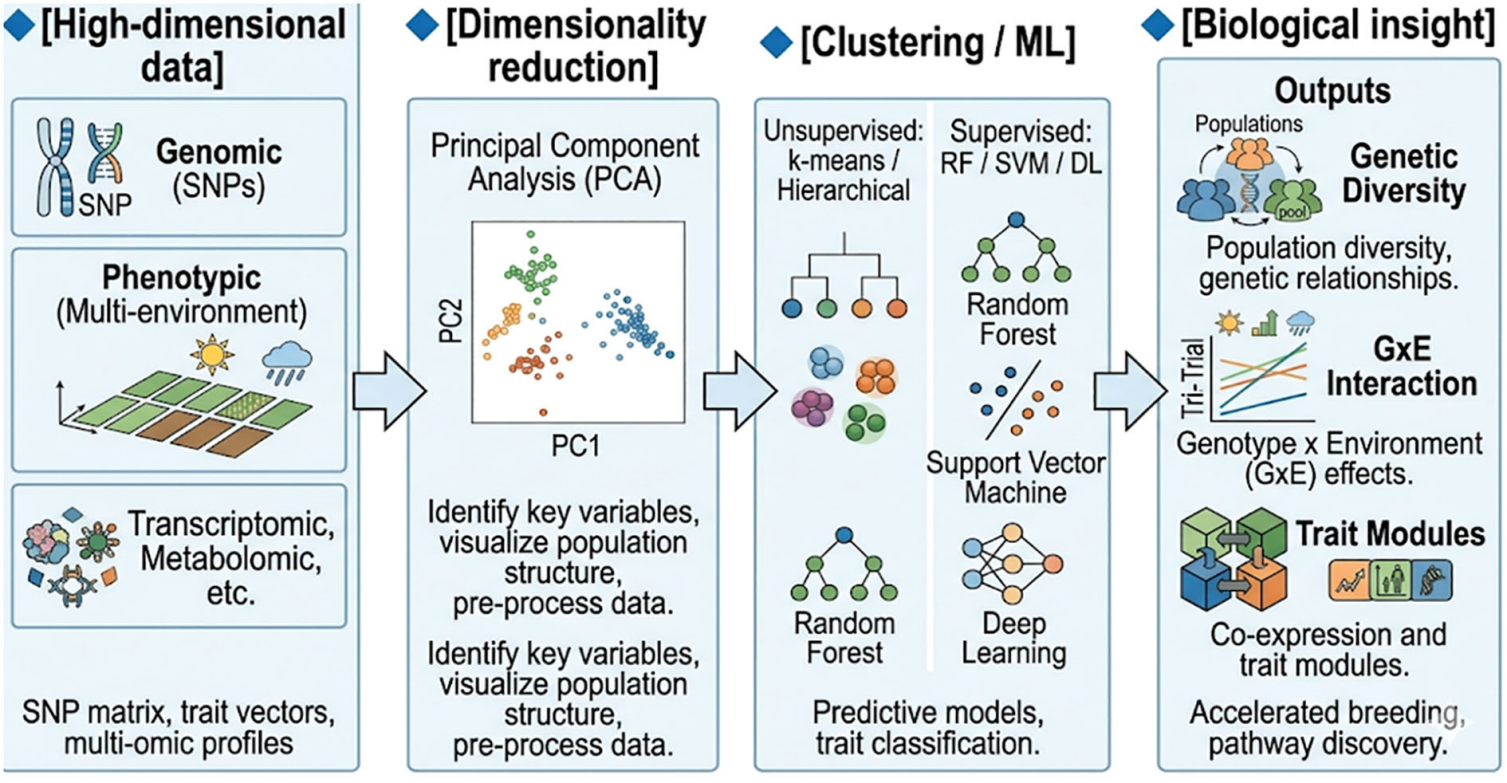
Conceptual framework of pattern recognition in plant breeding, highlighting the identification of data structures, clustering, and genotype-by-environment interactions to support selection decisions. Graphic design assistance provided by Gemini 3 Flash (Google).

Multivariate techniques such as PCA summarize high-dimensional variation and help visualize genotype clusters, population stratification, and relationships among traits or environments (**Figure 4**). Clustering methods, including hierarchical clustering and k-means, group genotypes or environments based on similarity, supporting germplasm management, parental selection, training population design, and environmental targeting (**Figure 4**). Factor analysis, correlation networks, and latent variable models can identify trait modules, trade-offs, and physiological or genetic relationships relevant to breeding objectives (Jolliffe and Cadima, 2016; Crossa et al., 2017).

Pattern recognition also supports genomic prediction and multi-environment modeling. Kernel methods and similarity-based approaches can capture nonlinear relationships among genotypes, while ML algorithms such as *RF*, *SVMs*, and deep learning can detect complex structures when sufficient data and appropriate validation are available (**Figure 4**). However, flexible models are vulnerable to overfitting, especially in “large p, small n” settings. Therefore, pattern discovery must be accompanied by cross-validation, biological interpretation, and uncertainty assessment.

The integration of multi-omics and environmental data further increases the importance of pattern discovery. Transcriptomic, metabolomic, phenomic, and enviromic datasets introduce additional layers of biological complexity. Statistical methods can help identify coherent modules, latent environmental gradients, and cross-layer relationships relevant for stress tolerance and adaptation. The practical goal is to convert hidden structure into actionable breeding information, such as identifying representative environments, selecting diverse parents, constructing balanced training populations, and improving the interpretation of prediction models.

### Resource optimization in plant breeding

**Figure 5:**
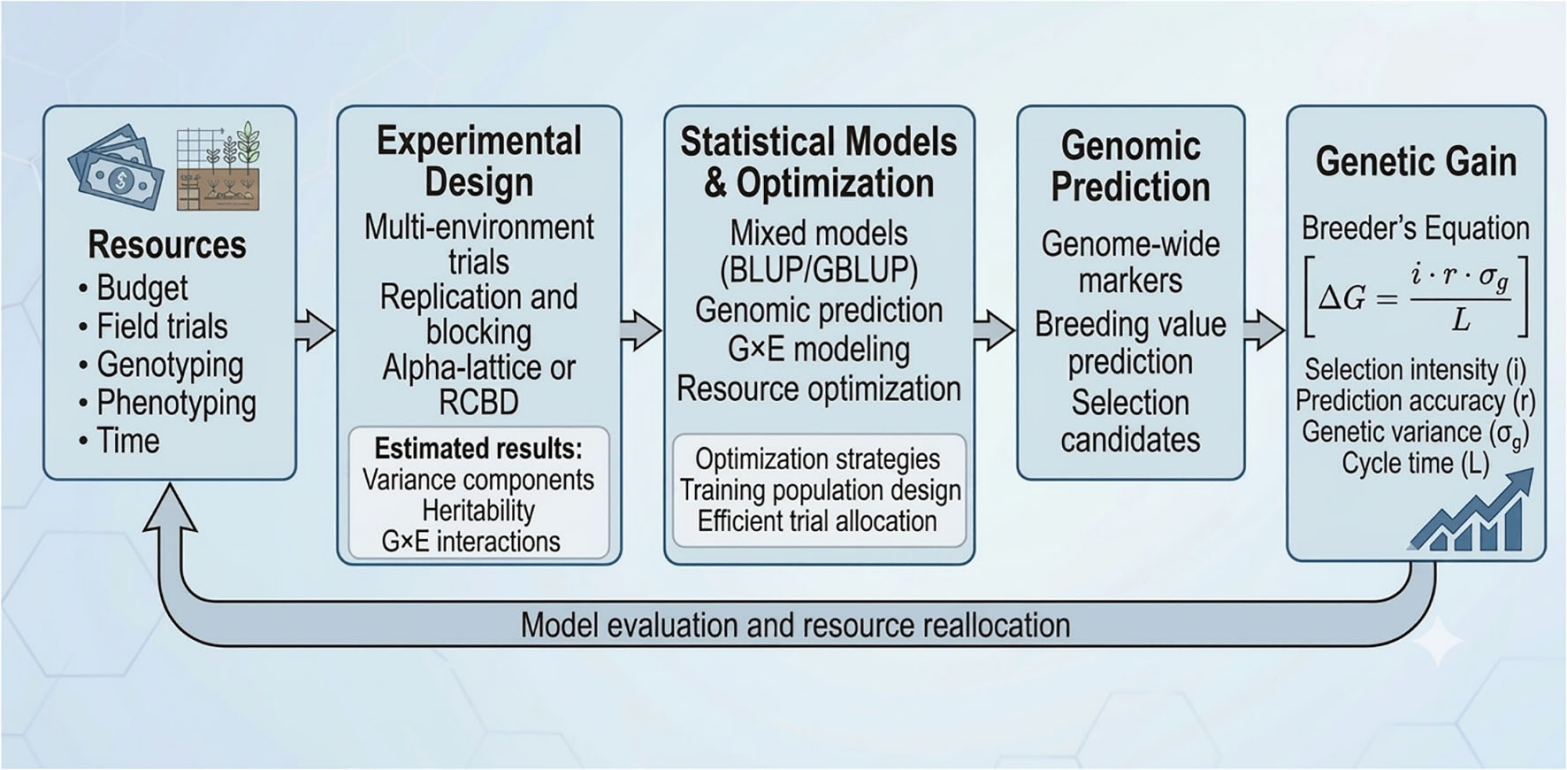
Conceptual framework for resource optimization in plant breeding. Statistical methods guide the allocation of resources across experimental design, genomic prediction, and multi-environment testing to maximize genetic gain. Experimental designs and mixed models enable estimation of variance components, heritability, and G×E interactions, while optimization strategies support training population design and efficient trial allocation. Genetic gain, as described by the breeder’s equation, depends on selection intensity, accuracy, genetic variance, and cycle time, and is iteratively improved through feedback between model evaluation and resource allocation. Graphic design assistance provided by Gemini 3 Flash (Google).

Statistics play a central role in optimizing resource use in plant breeding programs, where field trials, genotyping, phenotyping, and multi-environment testing are costly, labor-intensive, and often constrained by limited land, labor, time, and financial resources. A key component is experimental design, which determines how genotypes are allocated, replicated, randomized, and blocked to maximize precision and statistical power while minimizing costs. Classical and modern experimental designs, including randomized complete block designs, incomplete block designs, alpha-lattice designs, augmented designs, and row–column structures, help control spatial and environmental heterogeneity and improve the precision of estimated genetic effects (**Figure 5**) (Cochran and Cox, 1957; Federer, 1955; Patterson and Williams, 1976; Piepho et al., 2008).

These designs are closely linked to linear models, analysis of variance (ANOVA), and mixed models, which partition variation into genetic, environmental, genotype-by-environment (*G* × E), spatial, and residual components. Mixed-model methodology has become especially important in plant breeding because it allows the estimation of variance components, best linear unbiased predictions, heritability, and prediction error variances under complex experimental and multi-environment structures (Henderson, 1975; Searle et al., 1992; Piepho et al., 2008). By quantifying genetic variation and *G* × E effects, breeders can evaluate stability, identify informative environments, and determine the reliability of selection decisions.

Within genomic selection frameworks, resource optimization is critical for selecting training populations, identifying high-value phenotyping subsets, and allocating trials across environments. Because phenotyping remains one of the most expensive components of breeding programs, statistical criteria can be used to design training populations that maximize prediction accuracy while reducing unnecessary field evaluation (**Figure 5**). Approaches based on genomic relationships, coefficient of determination (CD), prediction error variance (PEV), expected reliability, and genetic algorithms help breeders select informative subsets of individuals for phenotyping and model training (Rincent et al., 2012; Akdemir et al., 2015; Isidro et al., 2015). These methods improve decisions about which genotypes should be evaluated, which populations should be prioritized, and how phenotyping and genotyping resources should be distributed (**Figure 5**).

Resource optimization is also grounded in the breeder’s equation, where genetic gain depends on selection intensity, prediction accuracy, genetic variance, and cycle time. Statistical and data science tools contribute to each of these components by increasing selection accuracy, accelerating breeding cycles, identifying optimal testing environments, and balancing short-term genetic gain with long-term diversity conservation (Falconer and Mackay, 1996; Lynch and Walsh, 1998; Jannink et al., 2010; Atlin et al., 2017). Thus, optimization methods are not merely computational exercises; they directly support breeder decisions about trial design, resource allocation, parent selection, genotype advancement, and breeding strategy.

Advanced approaches, including genetic algorithms, multi-objective optimization, simulation-based selection strategies, and optimal contribution selection, further enhance decision-making by allowing breeders to balance competing objectives such as genetic gain, cost efficiency, environmental adaptation, and maintenance of genetic diversity (**Figure 5**). These approaches are especially relevant in modern breeding programs that must integrate genomic, phenotypic, environmental, and multi-omics information while operating under resource constraints. Overall, resource optimization enables sustainable breeding progress by transforming limited inputs into maximal genetic improvement through rigorous statistical modeling and strategic decision-making.

### Testing hypotheses in plant breeding

**Figure 6:**
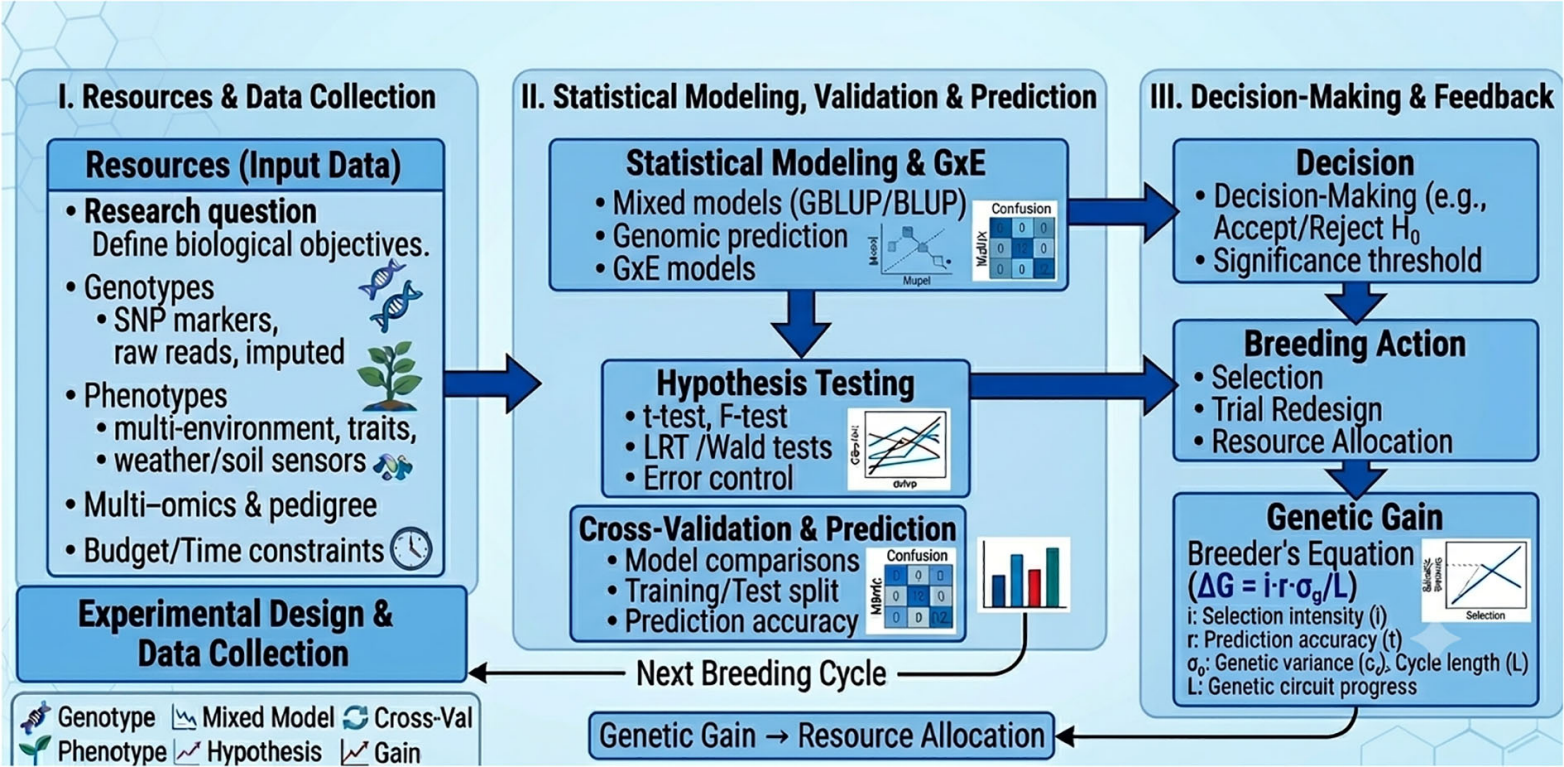
Integrated framework of hypothesis testing, model validation, and decision-making in plant breeding, illustrating the workflow from experimental design and statistical modeling to prediction, validation, and selection, ultimately supporting genetic gain and resource optimization. Graphic design assistance provided by Gemini 3 Flash (Google).

Hypothesis testing is a foundational statistical process in plant breeding because it helps determine whether observed differences among genotypes, treatments, environments, or model components reflect true biological signals or random variation. The process begins with a null hypothesis, typically stating no difference or no effect, and an alternative hypothesis reflecting the scientific or breeding question of interest. Classical tools such as t-tests, ANOVA, and linear mixed models have long supported genotype comparisons while accounting for experimental design, blocking, replication, and environmental variability (**Figure 6**) (Cochran and Cox, 1957; Federer, 1955; Searle et al., 1992; Piepho et al., 2008).

Modern plant breeding increasingly relies on mixed models that jointly incorporate fixed effects, such as treatments or environments, and random effects, such as genotypes, genetic relationships, spatial effects, and G × E interactions. These models provide a flexible framework for estimating variance components, predicting genetic values, and comparing genotypes across complex multi-environment trials (Henderson, 1975; Piepho et al., 2008). In this context, hypothesis testing supports practical breeding decisions by helping determine which genotypes should be advanced, which environments are most informative, and which sources of variation must be controlled to improve selection accuracy (**Figure 6**).

In GS, hypothesis testing is increasingly linked to model validation and decision-making. Likelihood-ratio, Wald, and score tests can be used to evaluate whether specific model components, such as environmental covariates, genomic relationship structures, dominance, epistasis, or G × E interaction terms, significantly improve model fit (**Figure 6**). Testing variance components can require special care because parameters may lie on the boundary of the parameter space, making standard asymptotic assumptions inappropriate in some cases (Self and Liang, 1987; Stram and Lee, 1994; Searle et al., 1992). Cross-validation and resampling-based procedures complement formal hypothesis testing by evaluating whether prediction accuracies are robust across populations, environments, or breeding cycles (Hastie et al., 2009; Jannink et al., 2010; de Los Campos et al., 2013).

In GWAS, hypothesis testing is central because thousands to millions of marker–trait associations are evaluated simultaneously. Multiple-testing procedures, including Bonferroni correction and false discovery rate (FDR) control, are essential to reduce false positives and support reliable biological interpretation (Benjamini and Hochberg, 1995; Yu et al., 2006; Korte and Farlow, 2013). Mixed-model association methods further improve inference by accounting for population structure and relatedness, thereby reducing spurious marker–trait associations. These inferential tools are essential for marker validation, candidate-gene prioritization, trait architecture interpretation, and marker-assisted selection (**Figure 6**).

However, modern breeding datasets introduce important challenges for hypothesis testing. Parametric assumptions such as normality, independence, and homoscedasticity may not always hold in genomic, phenomic, or environmental datasets. High-dimensional “large p, small n” settings, strong predictor correlations, population structure, environmental heterogeneity, and multi-omics integration complicate traditional testing frameworks. Resampling methods, permutation tests, model validation metrics, and Bayesian approaches can provide useful alternatives, but they also require careful interpretation and computational resources.

Despite these challenges, hypothesis testing remains essential for resource-efficient and scientifically credible decision-making. It helps prevent the advancement of inferior genotypes based on random noise, evaluates whether additional data layers genuinely improve prediction, and supports the interpretation of complex statistical models. In modern plant breeding, hypothesis testing should therefore be viewed not only as a formal inferential procedure, but as a decision-support tool that connects statistical evidence with practical breeding action.

### Computation of probabilities in plant breeding

Probability computation is fundamental for quantifying uncertainty and supporting informed decision-making under biological variability caused by segregation, recombination, environmental heterogeneity, sampling variation, and measurement error. Classical quantitative genetics is inherently probabilistic, relying on expectations and variances to describe breeding values, heritability, and response to selection (Falconer and Mackay, 1996; Lynch and Walsh, 1998). Rather than relying solely on point estimates, breeders use probabilities to evaluate the likelihood that a genotype exceeds a selection threshold, ranks among top candidates, or maintains superiority across environments.

Probability-based approaches are especially valuable in resource-limited contexts, early-generation selection, and multi-environment trials. Measures such as the probability of superiority, probability of correct selection, and predictive reliability allow breeders to prioritize genotypes based on both expected performance and associated uncertainty (Piepho et al., 2008). In mating design, Mendelian probabilities and recombination frequencies guide predictions about progeny genotype distributions and the likelihood of obtaining favorable allele combinations.

In GS, probability computation underpins the prediction of genetic merit and uncertainty around genomic predictions. Methods such as GBLUP and Bayesian genomic prediction generate predictive distributions or prediction error variances that quantify uncertainty around predicted genetic values (Meuwissen et al., 2001; Crossa et al., 2017). Bayesian models provide posterior distributions for marker effects and genomic values, while frequentist mixed models use prediction error variances to approximate selection reliability. These probabilistic outputs help breeders make risk-aware decisions rather than relying exclusively on deterministic rankings.

### Automation, reproducibility, and computational infrastructure

As breeding datasets become larger and more complex, automated and reproducible computational pipelines are increasingly important. From bioinformatics pipelines for omics data to GS and GWAS workflows, the installation and maintenance of multiple software tools can be difficult because of conflicting software dependencies. Container technologies such as Conda and Docker help integrate multiple algorithms and packages into reproducible environments that can be executed across operating systems and computing platforms (Kadri et al., 2022; Yu et al., 2024). Nevertheless, computational tools remain difficult for many users in the breeding chain, making accessible interfaces, online platforms, and training essential (Chen et al., 2024).

The use of graphics processing units (GPU) and parallel processing has also transformed big data analysis and deep learning in plant breeding. Unlike central processing units (CPU), which are optimized primarily for sequential processing, GPUs contain many smaller cores capable of executing multiple operations simultaneously. This parallel architecture can reduce training time and improve scalability for deep learning, image analysis, high-throughput phenotyping, and simulation-based analyses (Montesinos-López et al., 2024).

*TensorFlow* and *PyTorch* are widely used frameworks for implementing deep learning models, and both can exploit GPU acceleration through NVIDIA Corporation hardware and Compute Unified Device Architecture (CUDA). For users restricted to CPU environments, high-performance numerical libraries such as Intel Math Kernel Library (MKL) and Open Basic Linear Algebra Subprograms (OpenBLAS) can substantially improve computational performance. Traditional ML models, such as *SVMs* and *RF*, are commonly implemented in R and Python through packages such as scikit-learn, while gradient boosting algorithms such as Light Gradient Boosting Machine (*LightGBM)* and extreme gradient boosting (*XGBoost*) are widely used because of their computational efficiency (Bisong, 2019; Dai et al., 2022; Hill et al., 2024; Kolisnik et al., 2025; Zhang and Gong, 2020).

### Integration of pangenomes into GS and GWAS

The rapid evolution of sequencing technologies has enabled the generation of large numbers of high-quality genome assemblies. Pangenomics represents an important next step for GS and GWAS because it addresses limitations of single-reference genomes and captures structural variation, copy number variation, presence–absence variation, and transposable element diversity (Zhou et al., 2022). These sources of variation may contribute to missing heritability and may improve the biological interpretation of genomic analyses.

The integration of pangenomes into GS and GWAS requires graph-based representations, new algorithms, substantial storage capacity, and statistical methods capable of handling complex high-dimensional genomic structures (Jayakodi et al., 2025). For GWAS, pangenomic information may improve the detection of causal variants that are absent from a single reference genome. For GS, pangenome-derived markers and structural variants may enrich genomic relationship matrices or kernel models, potentially improving prediction for traits influenced by structural variation. However, the practical benefits will depend on data quality, population size, computational feasibility, and validation across breeding populations.

As with other data-intensive approaches, the value of pangenomics in breeding should ultimately be assessed by its contribution to better decisions. These include identifying useful allelic diversity, prioritizing germplasm from genebanks, improving candidate-gene discovery, enhancing prediction accuracy, and designing crosses that exploit both known and previously hidden genomic variation.

## Discussion

### Statistics and data science as the operational core of predictive breeding

The transformation of plant breeding into a predictive and decision-oriented discipline has redefined the role of statistics and data science. Rather than serving only as auxiliary tools for data analysis, these disciplines now provide the operational framework through which genetic variation is quantified, biological complexity is modeled, uncertainty is evaluated, and selection decisions are optimized. In this context, modern plant breeding can be understood as the integration of quantitative genetics, statistical modeling, computational data science, and breeding decision theory into a unified system that links biological processes with practical genetic improvement.

Quantitative genetics remains the biological and theoretical foundation of this system. Classical concepts such as genetic variance, heritability, breeding value, *G*×*E* interaction, and response to selection continue to guide how breeders understand complex traits and predict selection outcomes (Falconer and Mackay, 1996; Lynch and Walsh, 1998). However, the increasing availability of genomic, phenotypic, environmental, pangenomic, and multi-omics data has expanded the scale and complexity at which these principles must be implemented. Modern statistical and data science methods allow quantitative genetic theory to operate in high-dimensional settings where the number of predictors often exceeds the number of observations and where biological effects may be nonlinear, context-dependent, and distributed across many loci.

A central message of this review is therefore that advances in plant breeding should not be attributed to genomic technologies alone. Genomics provides the data, but statistics and data science provide the inferential, predictive, and computational machinery needed to transform these data into usable knowledge. GS, GWAS, multivariate analysis, mixed models, *ML*, hypothesis testing, probability computation, and optimization are not isolated methods; they are interconnected components of a predictive breeding pipeline. Their value lies in helping breeders make better decisions: which parents to cross, which genotypes to advance, which environments to target, which traits to prioritize, how training populations should be designed, and how limited resources should be allocated across phenotyping, genotyping, and multi-environment testing.

### From genetic architecture to predictive models

The performance of predictive breeding depends critically on how well statistical models represent the genetic architecture of complex traits. Classical linear models, including GBLUP, remain powerful and robust tools, particularly for traits governed mainly by many small additive effects. In such settings, linear genomic prediction models often provide stable performance, straightforward interpretation, and computational efficiency (Meuwissen et al., 2001; Jannink et al., 2010; Crossa et al., 2017). However, many agronomic traits are influenced not only by additive effects but also by dominance, epistasis, *G*×*E* interactions, and nonlinear relationships among genetic, environmental, and physiological factors.

This complexity motivates the use of more flexible models, including Bayesian whole-genome regression, kernel methods, reproducing kernel Hilbert space regression, random forests, support vector machines, and deep learning architectures (Heslot et al., 2012; Crossa et al., 2017; Manthena et al., 2022; Yoosefzadeh Najafabadi et al., 2023). These approaches can capture nonlinear relationships, interactions, and latent patterns that may not be adequately represented by simple additive models. Nevertheless, increased model flexibility does not automatically translate into better breeding decisions. Complex models can overfit, require larger training populations, be sensitive to tuning parameters, and reduce biological interpretability. Therefore, model choice should be guided by trait architecture, population size, prediction scenario, environmental diversity, validation strategy, and the practical decision being supported.

### Complementarity between GS and GWAS

Genomic selection and genome-wide association studies represent two complementary uses of genomic information. GS is primarily a prediction framework. Its objective is to estimate genetic merit or expected phenotypic performance for candidate genotypes, often before complete phenotypic evaluation is available. Its success depends on training population size, marker density, trait heritability, population structure, relatedness between training and prediction sets, environmental representation, and the validation design used to assess prediction accuracy (Jannink et al., 2010; de Los Campos et al., 2013; Crossa et al., 2017). For highly polygenic traits, GS is particularly valuable because it uses genome-wide information rather than relying only on markers that reach statistical significance.

GWAS, in contrast, is primarily an inference and discovery framework. It aims to identify genomic regions associated with phenotypic variation and to improve understanding of trait architecture. The reliability and resolution of GWAS depend on sample size, marker density, linkage disequilibrium, population structure, kinship, phenotypic quality, and environmental heterogeneity (Yu et al., 2006; Korte and Farlow, 2013; Gangurde et al., 2022). Mixed linear models have improved control of false positives by accounting for relatedness and structure, while multi-locus approaches such as FarmCPU can improve power and reduce confounding in high-dimensional association studies (Liu et al., 2016). However, GWAS results must be interpreted carefully because significant associations may explain only a small fraction of genetic variance, may be population-specific, and may not always transfer across environments or breeding populations.

The integration of GWAS and GS provides an opportunity to connect biological interpretation with predictive accuracy. Genomic regions identified by GWAS can be incorporated into GS models as fixed effects, weighted markers, biological priors, or kernel components. This integration may improve prediction and biological interpretability when traits are influenced by moderate- or large-effect loci. However, for highly polygenic traits, whole-genome prediction models that capture many small effects may remain more robust than approaches relying heavily on a limited number of significant markers. Thus, the value of integrating GWAS and GS depends on trait architecture, marker density, linkage disequilibrium, validation design, and the breeding decision being addressed.

### Data characterization, structure, and model reliability

Before applying GS, GWAS, or *ML* models, breeding datasets must be characterized in terms of quality, structure, relatedness, and dimensionality. Exploratory analysis, principal component analysis, clustering, genomic relationship matrices, heatmaps, residual diagnostics, and visualization tools are essential for identifying outliers, population structure, environmental patterns, and data-quality problems (Jolliffe and Cadima, 2016; Crossa et al., 2017). These procedures are not merely descriptive. They affect training population design, control of confounding in GWAS, interpretation of genomic relationships, selection of validation schemes, and the ability of models to generalize across populations and environments.

Population structure and relatedness are particularly important because they influence both inference and prediction. In GWAS, failure to account for structure can produce spurious associations. In GS, prediction accuracy often depends strongly on the genetic relationship between the training and prediction populations. Similarly, environmental heterogeneity and genotype-by-environment interactions can limit model transferability when prediction environments differ from training environments. Therefore, rigorous data characterization is a necessary step in transforming high-dimensional breeding data into reliable inference and useful prediction.

Visualization and pattern recognition also support breeder decision-making. Principal component analysis and clustering can help identify subpopulations, heterotic groups, genetic diversity, and potential parents. Heatmaps and dendrograms can reveal genotype or environment similarities. Spatial field maps and residual plots can identify experimental problems. Manhattan and quantile–quantile plots help evaluate GWAS results. Observed-versus-predicted plots and cross-validation summaries help evaluate prediction models. These tools contribute directly to decisions about which data are reliable, which environments are informative, which genotypes are representative, and which models are suitable for operational use.

### Prediction, inference, and optimization as a unified system

Modern breeding programs operate under constraints of time, land, labor, phenotyping capacity, genotyping cost, and environmental uncertainty. Therefore, prediction alone is not sufficient. Breeders must also decide how to allocate resources, how to design trials, how many genotypes to phenotype, which environments to sample, and how to balance short-term genetic gain with long-term diversity. Statistical design and optimization methods provide the framework for making these decisions more efficiently.

Experimental designs such as randomized complete blocks, incomplete blocks, alpha-lattice designs, augmented designs, and row–column structures improve the precision of genetic evaluation by controlling spatial and environmental heterogeneity (Cochran and Cox, 1957; Federer, 1955; Patterson and Williams, 1976; Piepho et al., 2008). Mixed models extend these designs by estimating genetic effects, variance components, genotype-by-environment interactions, and prediction error variances under complex multi-environment settings (Henderson, 1975; Searle et al., 1992; Smith et al., 2001; Piepho et al., 2008). These methods directly affect breeding decisions because they determine the reliability of genotype comparisons and the confidence with which superior material can be advanced.

In genomic selection, optimization is especially important for training population design and resource allocation. Methods based on genomic relationships, coefficient of determination, prediction error variance, expected reliability, and genetic algorithms can identify informative subsets for phenotyping and model training (Rincent et al., 2012; Akdemir et al., 2015; Isidro et al., 2015). Such approaches help breeders allocate phenotyping and genotyping resources where they produce the greatest improvement in prediction accuracy. More broadly, these methods contribute to the breeder’s equation by increasing selection accuracy, reducing cycle time, improving selection intensity, and helping maintain useful genetic variance (Falconer and Mackay, 1996; Lynch and Walsh, 1998; Jannink et al., 2010; Atlin et al., 2017).

### Uncertainty, hypothesis testing, and risk-aware decisions

Uncertainty is inherent in all breeding decisions because phenotypic observations are affected by genetic segregation, recombination, environmental variation, measurement error, and finite sampling. Statistical models are valuable not only because they provide point estimates or predictions, but because they quantify uncertainty. Prediction error variances, posterior distributions, confidence intervals, p-values, false-discovery rates, cross-validation distributions, and probabilities of superiority all help breeders evaluate risk and make more informed decisions (Gianola and Fernando, 1986; Piepho et al., 2008; de Los Campos et al., 2013).

Hypothesis testing remains important in modern plant breeding, even in prediction-oriented frameworks. In field trials and multi-environment testing, hypothesis testing helps determine whether observed differences among genotypes, environments, or treatments are likely to reflect true biological effects rather than random variation. In mixed models, likelihood-ratio, Wald, and score tests can be used to evaluate model components, although variance-component testing requires care because parameters may lie on the boundary of the parameter space (Self and Liang, 1987; Stram and Lee, 1994; Searle et al., 1992). In GWAS, multiple-testing correction and false-discovery-rate control are essential because thousands to millions of marker–trait associations are evaluated simultaneously (Benjamini and Hochberg, 1995; Yu et al., 2006; Korte and Farlow, 2013).

In GS and *ML*, cross-validation and resampling-based evaluation complement formal hypothesis testing by assessing whether prediction accuracy is robust across genotypes, environments, or breeding cycles (Hastie et al., 2009; Jannink et al., 2010; de Los Campos et al., 2013). This is particularly important when comparing models that differ in complexity or data inputs. A more complex model should not be adopted simply because it is more sophisticated; it should demonstrate consistent improvement in accuracy, robustness, biological interpretability, or decision value. Thus, hypothesis testing and validation are central to preventing overinterpretation and ensuring that statistical evidence translates into reliable breeding action.

### Interpretable and multimodal predictive systems

The future of predictive breeding will depend increasingly on the integration of multiple data modalities, including genomic, phenotypic, environmental, pangenomic, transcriptomic, metabolomic, and high-throughput phenotyping data. These data layers can provide complementary information about genetic potential, physiological status, environmental responses, and biological mechanisms. Multimodal and multi-kernel models, Bayesian frameworks, and deep learning architectures offer promising ways to integrate such heterogeneous information (Crossa et al., 2017; Montesinos-López et al., 2024; Yoosefzadeh Najafabadi et al., 2023).

However, multimodal data integration also introduces substantial challenges. Different data types may have different scales, missing-data patterns, noise structures, and biological relevance. Adding more data does not necessarily improve prediction, especially when sample size is limited or when additional covariates introduce noise. Therefore, future predictive systems must balance accuracy, biological interpretability, computational efficiency, and biological meaning. Interpretable *ML*, probabilistic modeling, causal inference, and biologically informed priors may help ensure that complex models remain useful for breeders rather than becoming opaque computational systems (Gelman et al., 2014; Pearl et al., 2016).

Pangenomics represents another important direction because it addresses limitations of single-reference genomes and captures structural variation, copy number variation, presence–absence variation, and transposable element diversity (Zhou et al., 2022; Jayakodi et al., 2025). These sources of variation may contribute to missing heritability and improve both GWAS and GS when structural variants are relevant to trait expression. Nevertheless, pangenomic integration will require scalable algorithms, graph-based representations, careful validation, and computational infrastructure capable of handling complex genomic data. As with other technologies, its value should ultimately be judged by its contribution to better breeding decisions, not only by the richness of the data it generates.

### Final perspective

The central contribution of statistics and data science to modern plant breeding is the transformation of quantitative genetics from a theoretical framework into an operational predictive and decision-making system. These disciplines enable breeders to estimate genetic effects, predict unobserved genotypes, identify genomic regions, characterize data structure, evaluate uncertainty, optimize resources, and make selection decisions under complex biological and environmental conditions.

However, the future of predictive breeding will not depend simply on adopting more complex models or generating larger datasets. It will depend on developing statistically rigorous, biologically meaningful, interpretable, and decision-oriented systems that help breeders act more effectively under uncertainty. In this sense, statistics and data science do not replace quantitative genetics. Rather, they extend its practical reach, unlock its predictive potential, and provide the tools needed to convert biological data into sustainable genetic gain.

## Conclusion

Statistics and data science have become central to modern plant breeding because they transform the theoretical principles of quantitative genetics into predictive, inferential, and decision-oriented tools. GS, GWAS, mixed models, multivariate analysis, *ML*, probability computation, hypothesis testing, and optimization should not be viewed as isolated analytical procedures, but as interconnected components of a breeding decision pipeline. Their main value lies in helping breeders make better decisions about parent selection, genotype advancement, trait prioritization, environmental targeting, training population design, resource allocation, and risk-aware selection under uncertainty.

Future progress will depend on developing integrated, interpretable, and scalable frameworks that combine genomic, phenotypic, environmental, pangenomic, and multi-omics information while maintaining a strong connection to biological meaning and practical breeding decisions. More complex models and larger datasets will be useful only when they improve prediction reliability, biological understanding, or decision quality. Thus, statistics and data science should not be seen as replacing quantitative genetics, but as extending its practical reach and unlocking its predictive potential for sustainable genetic improvement.

In conclusion, statistics and data science are not auxiliary components but *the conceptual and operational core of modern plant breeding*, enabling the transition from data to decisions and from observation to prediction. *Statistics has not replaced quantitative genetics—it has unlocked its full predictive potential.*
